# Accuracy and precision of four main glucometers used in a Sub-Saharan African Country: a cross-sectional study

**DOI:** 10.11604/pamj.2019.32.118.15553

**Published:** 2019-03-14

**Authors:** Simeon-Pierre Choukem, Colette Sih, Daniel Nebongo, Philomene Tientcheu, André-Pascal Kengne

**Affiliations:** 1 Department of Internal Medicine and Paediatrics, Faculty of Health Sciences, University of Buea, Buea, Cameroon; 2 Health and Human Development (2HD) Research Network, Douala, Cameroon; 3 Diabetes and Endocrine Unit, Department of Internal Medicine, Douala General Hospital, Douala, Cameroon; 4 UNILABO Biomedical Laboratory, Douala, Cameroon; 5 South African Medical Research Council and University of Cape Town, Cape Town, South Africa

**Keywords:** Cameroon, clinical accuracy, glucometer, precision, technical accuracy

## Abstract

**Introduction:**

capillary glucose measurement using point-of-care glucometers is an essential part of diabetes care. We determined the technical accuracy, clinical accuracy and precision of commonly available glucometers against standard spectrophotometry in Cameroon.

**Methods:**

a sample of four glucometers was selected. In the 108 diabetic and non-diabetic participants, blood glucose values obtained by glucometers were compared to the reference laboratory method to determine their technical and clinical accuracies. Precision was determined by repeated measurements using standard solutions of different concentrations.

**Results:**

accu-Chek^®^ Active, CodeFree™, Mylife™ Pura™ and OneTouch^®^ Ultra^®^ 2 values had correlation coefficients of 0.96, 0.87, 0.97 and 0.94 respectively with reference values, and biases of 18.7%, 29.1%, 16.1% and 13.8% respectively. All glucometers had ≥ 95% of values located within the confidence limits except OneTouch^®^ Ultra^®^2. Accu-Chek^®^ Active, CodeFree™, Mylife™ Pura™ and OneTouch^®^ Ultra^®^ 2 had 99%, 93.1%, 100% and 98.0% of values in Parke's zones A and B. The coefficients of variation of the glucometers were all below 5% at all standard concentrations, except for Accu-Chek^®^ Active for glucose concentrations at100 and 200mg/dL.

**Conclusion:**

no glucometer met all the international recommendations for technical accuracy. Accu-Chek™ Active and Mylife™, Pura™ met the International Organization for Standardization 2013 recommendations for clinical accuracy based on Parke's consensus error grid analysis. All glucometers assessed except Accu-Chek^®^ Active showed a satisfactory level of precision at all concentrations of standard solutions used.

## Introduction

The growing diabetes epidemics worldwide and in sub-Saharan Africa (SSA) poses diagnostic and management challenges [1]. Self-measurement and monitoring of blood glucose (SMBG) using point-of-care (POC) glucometers is an essential component of diabetic management and follow-up [2]. There is strong evidence that rigorous glycaemic control can in the long-run cost-effectively reduce the complications associated with type 2 diabetes [3]. The American Diabetes Association (ADA) recommends and promotes SMBG using glucometers in order to allow patients living with diabetes to achieve and maintain desired glycaemic targets [4]. Glucometers are very useful in timely diagnosis of acute diabetes complications, especially hypoglycaemia and hyperglycaemia which can contribute to higher morbidity and mortality [5]. Owing to the wide acceptance and use of POC glucometers in different settings, there is a competition-driven development by manufacturers in both meter and test strips technology. These innovations have allowed for greater reliability of results when compared to the reference laboratory method. Nonetheless, there are differences in performance across these monitoring devices. These have prompted the need to develop standard performance guidelines to guide manufacturers on the minimum requirements for these glucometers [6-8]. The revised performance guidelines published by the International Standardisation Organisation (ISO) 15197:2013 stipulate in the new criteria that for glucose values above 100mg/dL, the accuracy is expected to increase from ±20% to ±15% of the reference value; and this shall apply to at least 95% of results [6]. Many recent studies have highlighted the limitations of most glucometers in meeting these recommendations [9-11], very few of which were carried out in SSA [12]. Because of limited resources in most SSA countries, glucometers are used beyond their intended purpose, including for diabetes diagnosis for instance. Inaccurate glucometers would therefore negatively impact on decision making and downstream consequences more in SSA than elsewhere. Furthermore, uncontrolled competition and weak or inexistent standard quality control procedures are likely to favour the marketing of sub-standards glucometers in SSA than anywhere else. Lastly, it has been shown that climate conditions can affect the performance of glucometers [10]. In the current study, we assessed the technical and clinical accuracy and precision of glucometers commonly used in Cameroon.

## Methods

**Study design, setting and population:** in this cross-sectional study carried out from 1^st^ November 2014 to 28 February 2015, participants were recruited at the Douala General Hospital (DGH), whilst laboratory analysis of venous plasma glucose was carried out at UNILABO^®^ biomedical laboratory in Douala. Douala is the economic capital of Cameroon. The Douala General Hospital is a reference health institution, which provides a wide array of services, including a diabetic clinic. Diabetic patients attending the diabetic clinic at the Douala General Hospital as well as healthy volunteers (invited by advertisement) who provided written informed consent were eligible to participate. Participants with the following characteristics were excluded: hypotension with a systolic blood pressure of less than 90 mmHg, clinical anaemia, history of gout, history of jaundice and current anticoagulant therapy. Ethical approval was obtained from the Institutional Review Board of the Faculty of Health Sciences, University of Buea. Administrative clearance was obtained from the Director of the Douala General Hospital. All participants provided written informed consent.

**Selection of glucometers:** a survey of glucometers commercially available in Cameroon was conducted by visiting 5 randomly selected pharmacies in each of the ten regional headquarters of the country. Glucometers with complete system components were eligible to be included in the study. We excluded 6 glucometers for which pharmacies did not have sufficient number of strips (at least 150) per glucometer required for the study. The 4 glucometers finally evaluated were randomly purchased in the cities of Bamenda, Buea, Douala and Yaoundé: Accu-Chek^®^ Active (Roche Diagnostics GmbH, Mannheim, Germany), CodeFree™ (SD Biosensor Inc., South Korea), Mylife™Pura™ (Ypsomed AG, Burgdorf, Switzerland) and OneTouch^®^Ultra^®^2 (LifeScan Inc, USA).

**Study procedure and data collection:** for each consenting eligible participant, data were collected on age, sex and diabetes status. Participants were asked to wash their hands with tap water and neutral soap. A capillary sample was obtained from the middle finger. Blood was dropped in a systematic manner on the test strips of each glucometer and the results recorded. The glucometers were rotated so that no glucometer always occupied the same position. Concomitantly, venous whole blood samples were collected from the right or left forearm into 4mL sodium fluoride/oxalate tubes. The tubes were then appropriately labelled and stored at room temperature, transported to the laboratory for plasma glucose measurement within 120 minutes. In the laboratory, samples were centrifuged and venous plasma glucose obtained using the hexokinase enzymatic method in a Roche-Hitachi Cobas C111^®^ analyser (Roche Diagnostics GmbH, Mannheim, Germany; Hitachi High-Technology Corporation, Tokyo, Japan). The laboratory value was used as a reference for comparison. Every morning, calibration of the automated analyser was carried out by the same laboratory technician. Calibration of the glucometers using test strips was carried out daily. To avoid any bias, neither the technician doing laboratory analysis, nor the investigator carrying out measurements with glucometers was aware of each other results until the end of the study. Finally, we carried out repeated measurements of glucose concentrations on standard solutions of varying concentrations on each glucometer over one day. The standard solutions were prepared at the Chemistry Laboratory of the Faculty of Sciences, University of Buea. The concentrations of the standards were: 100 mg/dL, 200 mg/dL and 300 mg/dL. Each of the standard solutions was tested five times on each glucometer.

**Data analysis:** data analysis was carried out using the R statistical software V.3.1.1 (The R Foundation for Statistical Computing, Vienna, Austria). Continuous variables are reported as mean (and standard deviation, SD) or median (and 25^th^-75^th^ percentiles). Correlation between reference laboratory and each glucometer measurement was determined via covariance estimation for multivariate t distribution, which is known to be robust to the effect of outliers. Robust linear regressions methods were then used to derive the regression coefficients for the regression curve for predicting the reference values from the glucometer values. Paired-sample t-test was used to compare the mean difference between reference values and glucometer values; then the percentage bias was calculated as follows: (glucometer reading-reference value) × 100 ÷ reference value [13]. This was compared to the American Diabetes Association (ADA) standard of a bias of < 5% being acceptable [14, 15]. The agreement between the two measurements (that is, the glucometer and reference readings) at any given level was then examined using Bland-Altman plots [16]. Glycaemic values were also evaluated in the light of ISO 2013 requirements for accuracy. A p value of < 0.05 was considered statistically significant. To assess the clinical accuracy, we used the total error allowable (TEa) and the Parke's consensus error grid analyses. The TEa was used to determine the clinical significance of differences observed between glucometer results and the reference. The mean of each glucometer reading was compared with the reference laboratory value and should be within clinical range of reference mean±TEa. Parke's error grid analysis is based on the comparison of clinical consequences of using the POC glucometer testing vs. the reference method. The analysis plots the glucometer-measured glucose against reference glucose level, into 5 zones: zone A (results given by the glucometer allow for clinically correct management decisions to be taken either in the hypoglycaemic or hyperglycaemic range), zone B (there is a deviation of >20% of glucometer results from the reference method. It represents values that would lead to benign or no treatment error.), zone C (results given by the glucometer would begin to lead to treatment decisions opposite to those based on reference blood glucose levels), zone D (results given by the glucometer lead to a failure to detect and treat errors) and zone E (glucometer-generated results fail to identify hypoglycaemia or hyperglycaemia. Values given by the glucometers are opposite to the reference values resulting in corresponding treatment decisions opposite to those needed) [15, 17, 18]. For perfect accuracy, 95% of values should be in zone A, 5% in zone B, and 0% in other zones. The precision of glucometers was determined by the calculation of the coefficients of variation (CV) that was further compared to the ISO criteria. The CV was computed as the standard deviation divided by the mean and expressed as a percentage [13]. A CV of less than 5% was considered as being precise [19].

## Results

**General characteristics of participants:** of the 108 study participants (53.7% women) included, six had missing readings for at least one glucometer. Therefore, only values from 102 participants with valid results recorded on all four glucometers were included in the analyses where the four glucometers were compared. The 108 participants' age ranged from 19 years to 81. The mean (standard deviation, SD) age was 46.2 (15.6) years. Participants with diabetes represented 68.5% (74.1% of women, 62.0% of male).

**Figure 1:**
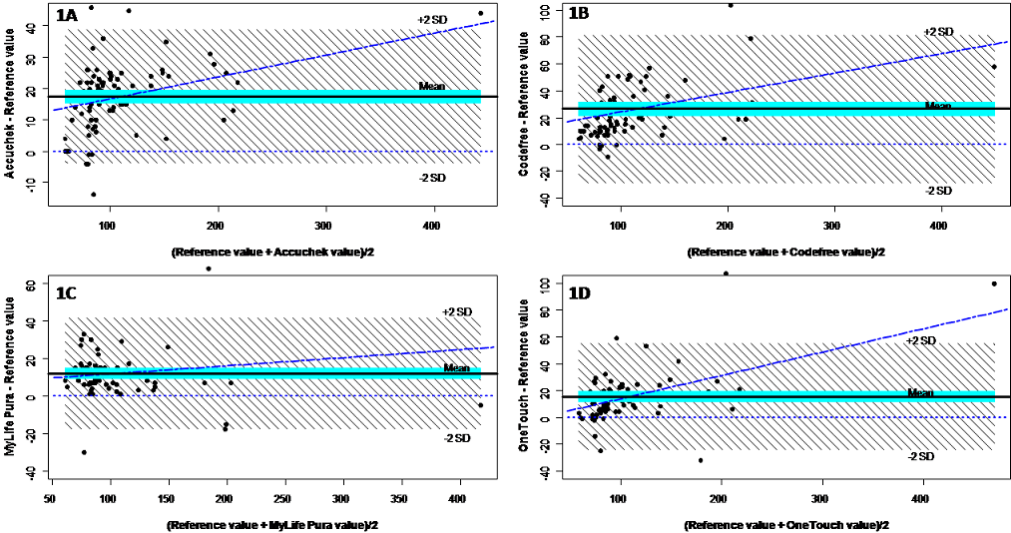
Bland-Altman plots for agreement between glucometers; (A) Accu-Chek^®^ Active; (B) CodeFreeTM; (C) MylifeTMPuraTM; (D) OneTouch^®^ Ultra^®^2) and the reference method, glucometer values minus reference value for each participant (y-axis) is plotted against the mean of the two measurements (x-axis) and are represented by the black dots; the horizontal doted blue line through zero is the line of perfect agreement between the two measurements; the parallel solid black line is the mean bias; the green colour band around the solid black line is the standard error around the mean bias estimates, while the shaded area represents the 95% confidence intervals; the linear curve of best fit is also shown (broken oblique blue line)

**Technical accuracy of glucometers:** the values provided by the glucometers were significantly higher than the reference; the mean differences and mean biases between glucometer results and the reference varied from +12.0 and 13.8% respectively for OneTouch^®^ Ultra^®^2 to +26.5 and 29.1% respectively for CodeFreeTM (Table 1). There was a significant positive correlation between each glucometer and the reference values, with correlation coefficients ranging from 0.87 (95%CI 0.81-0.91) for CodeFree^TM^ and 0.97 (0.95-0.98) for Mylife™ Pura™ (Table 2). For all glucometers, the correlation coefficients were always higher in men than women (all p < 0.01 for correlation coefficient comparisons; the linear regression allowed determination of the equation linking the reference blood glucose to each glucometer's blood glucose (Table 2). According to the ISO recommendations of 2013, 95% of values are expected to fall within the confidence limit for agreement in the Bland-Altman plots to be considered as being acceptable. Bland-Altman plots showed 5 outliers (4.9%) for Accu-Chek^®^ Active with 4 in the positive region and 1 in the negative region (Figure 1A), 2 outliers (1.96%) for CodeFree^TM^ both being in the positive region (Figure 1B), 1 outlier (0.98%) in the negative region for Mylife™ Pura™ (Figure 1C). Hence, there was a good agreement between the values generated by the reference method and these glucometers, whereas the agreement was lower for OneTouch^®^ Ultra^®^2 as there were 6 outliers (5.88%), with 4 in the positive region and 2 in the negative region (Figure 1D). Based on ISO 2013 recommendations, no glucometer met the criteria for the required level accuracy of 99%. The lowest proportion was with CodeFree™ in the range of glycemia < 100mg/L and Accu-Chek^®^ Active in the range of glycemia > 100mg/dL, whereas Mylife™ Pura™ yielded the highest proportion within the two ranges (Table 3).

**Table 1:** absolute difference between each glucometer and reference method results

Glucometer	Mean difference	95% CI	Mean bias (%)	P
Accu-Chek^®^ Active	+17.4	15.4 - 19.5	18.7	<0.0001
CodeFree^TM^	+26.5	21.3 - 31.8	29.1	<0.0001
Mylife^TM^Pura^TM^	+15.5	11.7 - 19.3	16.1	<0.0001
OneTouch^®^ Ultra^®^2	+12.0	9.2 - 14.9	13.8	<0.0001

CI: confidence interval

**Table 2:** correlations between the reference value and glucometer-based values,and regression equations

Glucometer	Overall (95% CI)	Women (95% CI)	Men (95% CI)	p*	Regression equation
Accu-chek^®^ Active	0.96 (0.94-0.97)	0.93 (0.88-0.96)	0.97 (0.95-0.98)	0.03	0.9117×AccuChek - 6.9173
CodeFree^TM^	0.87 (0.81-0.91)	0.73 (0.57-0.83)	0.95 (0.92-0.97)	<0.001	0.85690×CodeFree - 4.52115
Mylife^TM^Pura^TM^	0.97 (0.95-0.98)	0.94 (0.90-0.97)	0.98 (0.96-0.99)	0.01	1.05663×MyllifePura- 15.98433
OneTouch^®^ Ultra^®^2	0.94 (0.91-0.96)	0.87 (0.79-0.92)	0.96 (0.94-0.98)	<0.001	0.90853×OneTouch- 2.93158

* Women vs. men; CI: confidence interval

**Table 3:** proportion of values respecting ISO 2013 criteria for each glucometer

GLUCOMETER	≤ 100 mg/dL	>100 mg/dL
Accu-Chek^®^ Active	34/75 (45.3%)	14/32 (43.8%)
CodeFree^TM^	34/76 (44.7%)	16/32 (50.0%)
Mylife^TM^Pura^TM^	54/76 (71.1%)	27/30 (90.0%)
OneTouch^®^ Ultra^®^2	49/75 (65.3%)	20/30 (66.7%)

**Figure 2:**
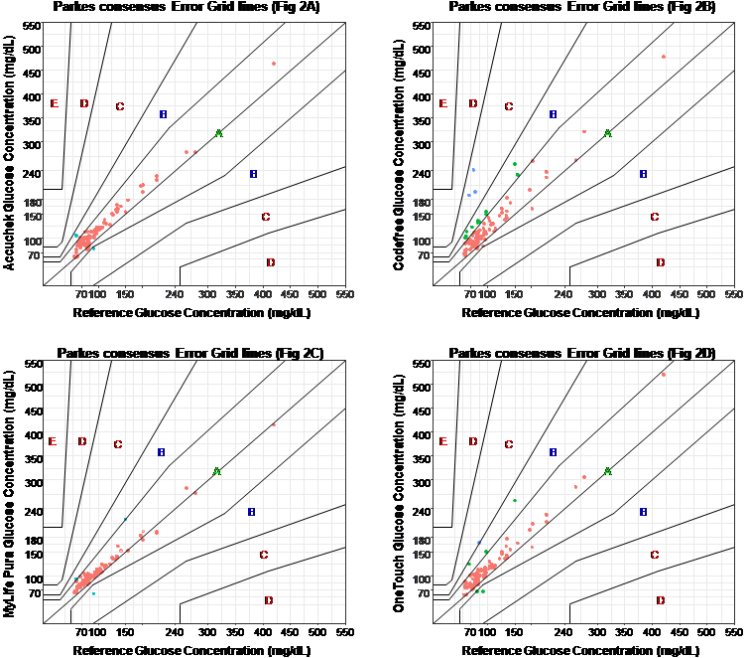
Parke’s consensus error grid analysis for glucometers; (A) Accu-Chek^®^ Active; (B) CodeFreeTM; (C): MylifeTMPuraTM; (D): OneTouch^®^ Ultra^®^2), Zone A: results given by the glucometer are clinically accurate within+/-20% of the reference; zone B: the error of the results given by the glucometer is above 20%, but would lead to minor or no treatment error; zone C: results given by the glucometer would begin to lead to treatment decisions opposite to that called for by the real blood glucose levels; zone D: results given by the glucometer lead to a failure to detect and treat errors; zone E: results given by the glucometer lead to erroneous treatment of hypo or hyperglycemia

**Clinical accuracy of glucometers:** all differences between the reference value and those provided by the various glucometers were both statistically significant (p < 0.0001) as well as clinically significant, without any gender difference. According to Parke's consensus error grid analysis, Accu-Chek^®^ Active had 99% (n = 101) of its values within zones A and B and 1% (n=1) in zone C (Figure 2A), CodeFree™ had 93.1% (n=95) of its values in zone A and B, 4.9% (n=5) in zone C and 2% (n=2) in zone D (Figure 2B). Mylife™ Pura^TM^had 100% of its values in zone A and B (Figure 2C) and OneTouch^®^ Ultra^®^2 had 98.0% (n=100) in zone A and B and 2% (n=2) in zone C (Figure 2D). Based on ISO recommendations of 2013, Accu-Chek^®^ Active and Mylife™ Pura™ met the criteria, with 99 to 100% of their values found within zones A and B; OneTouch^®^ Ultra^®^2 (98%) was close to the normal limit, whereas CodeFree^TM^(93.1%) fell short of the recommendations.

**Precision of glucometers** ACCU-CHEK^®^ Active provided an acceptable precision (CV < 5%) only for the high standard concentrations, whereas CodeFree™ Mylife™ Pura™ and OneTouch^®^ Ultra^®^2 all had acceptable level of precision at all concentrations of standard solutions.

## Discussion

We have shown in this study that none of the glucometers tested consistently met all the international recommendations set by the ISO and the ADA for technical accuracy. Although none met the criteria for clinical accuracy based on TEa, Accu-Chek^®^ Active and MylifeTMPuraTM met the ISO 2013 requirements based on Parke's consensus error grid. All glucometers except Accu-Chek^®^ Active showed a satisfactory level of precision at all concentrations of standard solutions used. Many other studies have also reported that not all glucometers meet the requirements. In a study carried out in South Africa in 2009 [9] five glucometers were assessed: GlucoPlus^TM^ (Diabcare), OneTouch^®^ Ultra^TM^ (LifeScan Inc, Johnson & Johnson), OneTouch^®^ Horizon^TM^(Johnson & Johnson), Accu-Chek^®^ Active (Roche) and Accu-Chek^®^ Advantage (Roche) on 115 diabetic patients. Generally, only three glucometers (GlucoPlus^TM^, OneTouch^®^ Horizon^TM^ and Accu-Chek^®^ Active) met the ISO 2003 guidelines whereas none met the ADA requirements. Most other studies that included similar glucometers (Accu-Chek^®^ Active and OneTouch^®^ Ultra^®^2) have yielded results with some similarities, but also discrepancies with our findings at various levels of accuracy [20-22]. We have not found published studies that have evaluated CodeFree^®^. Amongst the four glucometers evaluated in our study, two (ACCU-CHEK^®^ Active and mylife^TM^Pura^TM^) had the ConformitéEuropéenne (CE) label in 2010 [23]. In the study by Freckmann *et al*. the conformity of 27 glucometers bearing the CE label was evaluated using 100 blood samples, with a defined distribution of blood glucose concentrations ranging from 20 mg/dL to 600 mg/dL [23]. Only 16 out of the 27 glucometers met the minimum ISO 2003 requirements. Amongst these were Accu-Chek^®^ Active and Mylife^TM^Pura^TM^, with 100% of the values provided by both glucometers respecting the ISO 2003 recommendations. They used similar methods in 2012 [25] to assess 43 glucometers with the CE label, using 100 capillary samples spanning across concentrations of 50 to 400 mg/dL. Amongst these, only 34 were completely evaluated and 27 showed an acceptable level of accuracy based on ISO 2003 recommendations, amongst which Accu-Chek^®^ Active and Mylife^TM^Pura^TM^. The lower accuracy observed in our study suggests the potential alteration of glucometers or strips by climate conditions after a long stay in SSA, as suggested earlier [10].

Also, whether glucometers of lower standards are manufactured and marketed in our environment in the absence of rigorous quality control standards is unknown. Even though POC glucometers are not intended for screening and diagnosis of diabetes based on guidelines provided by the FDA, [7, 8] these devices are nonetheless used for screening and diagnosis of diabetes in SSA. All the glucometers assessed during our study overestimated the blood glucose concentration. Hence, the clinical implication of using any of these glucometers in the screening and diagnosis of diabetes is that they correctly identify patients with diabetes, but misdiagnose individuals with borderline normal or impaired fasting glucose as having impaired fasting glucose or diabetes, respectively. With regards to the use of the glucometers evaluated in our study as tools for monitoring, there might be a greater risk of iatrogenic hypoglycaemia especially with CodeFree^TM^, because they may result in the use of higher doses of hypoglycaemic agents. Also, the glucometers may fail to identify hypoglycaemia due to overestimation of blood glucose levels. Regarding the statistically significant difference between women and men in the correlation between reference values and values provided by all glucometers, we speculate that they may be due to the influence of body creams and lotions used by Cameroonian women, which may contain contaminants that interact with capillary glucose measurement [26]. The following potential limitations of our study should be considered when interpreting and generalizing our findings. Firstly, ISO recommendations were meant for self-testing. Hence, the assessment of the accuracy by a medically trained person can potentially overestimate the accuracy of the glucometers when compared to self-testing. Secondly, although all participants carefully washed their hands before capillary glucose measurement, all potential interferences to glucometer readings may not have been eliminated. Also, the investigator performing capillary glucose measurements was not blinded to the meter being used. Nevertheless, the reference values were made available only after the completion of all glucometer-based glucose determination. Finally, measurements in the hypoglycaemic range could not be carried out; hence, performance of the glucometers could not be assessed at all glycaemic ranges. Despite these potential limitations and unlike other studies that have been carried out on glucometers, we used very exhaustive analytical methods and endeavoured to compare the findings of our study to recommendations of more than one international body.

## Conclusion

In conclusion, based on the extensive comparisons conducted in this study, no glucometer met all the required international recommendations; hence, all glucometers should always be used with caution, especially if their results mandate serious clinical decision. However, Mylife^TM^Pura^TM^ would be the best recommended glucometer in Cameroon for screening and follow-up of diabetes. Despite its satisfactory precision, CodeFree^®^ seems not to be recommended because it showed consistent technical and clinical inaccuracies.

### What is known about this topic

- It is known from studies conducted in other countries across the world that point-of-care glucometers do not always meet the international requirements for performance;
- Failure of glucometers to comply with the norms will lead to erroneous management of diabetic patients.

### What this study adds

- This study is one of the first of its kind in Cameroon that seeks to determine the accuracy and precision of four commonly used glucometers in Cameroon when compared to reference laboratory method;
- This study gives suggestions based on our results for which glucometers might be better for Cameroon.

## Competing interests

The authors declare no competing interests.
